# Mortality, Stroke, and Hospitalization Associated With Deferred vs Expedited Aortic Valve Replacement in Patients Referred for Symptomatic Severe Aortic Stenosis During the COVID-19 Pandemic

**DOI:** 10.1001/jamanetworkopen.2020.20402

**Published:** 2020-09-30

**Authors:** Christoph Ryffel, Jonas Lanz, Noé Corpataux, Nicole Reusser, Stefan Stortecky, Stephan Windecker, Thomas Pilgrim

**Affiliations:** 1 Department of Cardiology, Inselspital, Bern University Hospital, University of Bern, Bern, Switzerland

## Abstract

This cohort study evaluates the outcomes associated with deferred vs expedited aortic valve replacement in patients with severe aortic stenosis during the COVID-19 pandemic.

## Introduction

The coronavirus disease 2019 (COVID-19) pandemic represents an unprecedented challenge for health care systems and requires the redistribution of health care resources within hospitals. Between March 20 and April 26, 2020, the Federal Council of Switzerland banned elective procedures in all hospitals in Switzerland.^1^

Balancing the risk of deferral of treatment of symptomatic severe aortic stenosis vs expedited aortic valve replacement (AVR) during the time of health care restrictions requires careful clinical judgement. The aim of the present study was to prospectively evaluate outcomes in a cohort of patients with symptomatic severe aortic stenosis who received deferred vs expedited AVR based on prespecified criteria during the COVID-19 pandemic.

## Methods

In this cohort study, the Aortic Stenosis Defer (AS DEFER) study, all patients with symptomatic severe aortic stenosis referred for AVR between March 20 and April 26, 2020, were consecutively included. Definitions, data collection, end point ascertainment, statistical analysis, and study flow are detailed in the eAppendix in the Supplement.^2^ This study was approved by Ethics Committee Bern, Switzerland. All patients provided written or oral informed consent for participation in this study. This study followed the Strengthening the Reporting of Observational Studies in Epidemiology (STROBE) reporting guideline.

Patients with critical aortic stenosis defined by an aortic valve area of 0.6 cm^2^ or less, a transvalvular mean gradient of at least 60 mm Hg, cardiac decompensation during the previous 3 months, or exercise intolerance with clinical symptoms on minimal exertion were allocated to expedited AVR. Patients with an aortic valve area of 1.0 cm^2^ or less and greater than 0.6 cm^2^ and stable symptoms were allocated to deferred AVR. The primary end point was a composite of all-cause mortality, disabling and nondisabling stroke, and unplanned hospitalization for valve-related symptoms or worsening heart failure by intention to treat. A 2-sided *P* < .05 was considered statistically significant. Statistical analyses were performed using SAS software, version 9.4 (SAS Institute).

## Results

Between March 20 and April 26, 2020, a total of 71 patients with symptomatic severe aortic stenosis were prospectively enrolled in the study. According to the prespecified algorithm, 25 patients (35.2%) were allocated to expedited AVR and 46 patients (64.8%) were allocated to deferred AVR. Baseline characteristics are summarized in the Table. All patients allocated to expedited AVR underwent transcatheter AVR at a mean (SD) of 10 (10) days after referral; none of the patients underwent surgical AVR.

**Table.** Baseline Clinical Characteristics of the Study Sample^a^

Characteristic	Patients		*P* value^b^
	Deferred AVR (n = 46)	Expedited AVR (n = 25)	
Age, mean (SD), y	77.2 (7.8)	79.6 (6.7)	.18
Female	22 (47.8)	10 (40.0)	.62
BMI, mean (SD)^c^	27.2 (5.2)	28.2 (4.2)	.46
Symptoms			
NYHA class III or IV^d^	13 (29.6)	17 (68.0)	.003
CCS grade III or IV	1 (2.2)	1 (4.0)	>.99
Syncope	4 (8.7)	1 (4.0)	.65
COVID-19 assessment			
Fever^e^	0	1 (4.0)	.42
Cough^e^	3 (6.5)	1 (4.0)	.63
Sore throat^f^	0	0	NA
SARS-CoV-2 PCR test^g^	6 (13.0)	12 (48.0)	.02
Risk assessment: STS-PROM score, mean (SD)^b^	3.3 (2.6)	2.9 (2.0)	.59
Medical conditions			
Diabetes	14 (30.4)	9 (36.0)	.79
Hypercholesterolemia	28 (60.9)	11 (44.0)	.21
Hypertension	41 (89.1)	23 (92.0)	>.99
Coronary artery disease	14 (30.4)	8 (32.0)	>.99
COPD	1 (2.2)	1 (4.0)	>.99
GFR <60 mL/min	19 (41.3)	10 (40.0)	>.99
History of atrial fibrillation	9 (19.6)	9 (36.0)	.16
Previous stroke or TIA	2 (4.3)	4 (16.0)	.17
Echocardiography findings			
Aortic valve, mean (SD)			
Gradient, mean (SD), mm Hg	40.6 (13.3)	50.5 (13.2)	.003
Area, cm^2^^d^	0.8 (0.2)	0.7 (0.2)	.11
LVEF, mean (SD), %	57.9 (14.5)	58.9 (13.1)	.78
Multivalvular heart disease	7 (15.2)	1 (4.0)	.24
Mitral regurgitation, moderate or severe	7 (15.2)	1 (4.0)	.24
Tricuspid regurgitation, moderate or severe	2 (4.3)	0	.54
AVR			
Transcatheter	4 (8.7)	25 (100)	<.001
Surgical	3 (6.5)	0	.55

Abbreviations: AVR, aortic valve replacement; BMI, body mass index (calculated as weight in kilograms divided by height in meters squared); CCS, Canadian Cardiovascular Society; COPD, chronic obstructive pulmonary disease; GFR, glomerular filtration rate; LVEF, left ventricular ejection fraction; NA, not applicable; NYHA, New York Heart Association; PCR, polymerase chain reaction; SARS-CoV-2, severe acute respiratory syndrome coronavirus 2; STS-PROM, Society of Thoracic Surgery predicted risk of mortality; TIA, transient ischemic attack.

^a^ Data are presented as number (percentage) of patients unless otherwise indicated.

^b^ *P* values were derived from the Fisher exact test for categorical variables and the *t* test for continuous variables.

^c^ Data were not available for 6 patients.

^d^ Data were not available for 2 patients.

^e^ Data were not available for 2 patients.

^f^ Data were not available for 21 patients.

^g^ Data were not available for 14 patients.

At a mean (SD) duration of follow-up of 31 (11) days after treatment strategy allocation, the primary composite end point occurred in 9 patients (19.6%) in the deferred treatment arm and in 1 patient (4.0%) in the expedited treatment arm (log rank *P* = .08) (Figure). Hospitalizations for valve-related symptoms or worsening heart failure were more common in patients allocated to deferred AVR compared with expedited AVR (19.6% vs 0%, *P* = .02). Patients allocated to deferred AVR who required hospitalization for valve-related symptoms or worsening heart failure more commonly had multivalvular disease (44.4% vs 8.6%, *P* = .02). Seven patients (15.2%) hospitalized for valve-related symptoms or worsening heart failure crossed over to expedited transcatheter AVR (n = 4) or surgical AVR (n = 3) within a mean (SD) of 17 (11) days after interdisciplinary allocation of the treatment strategy. One patient allocated to expedited transcatheter AVR experienced a periprocedural nondisabling stroke; none of the patients died. Compared with patients with no event, patients who experienced a primary outcome–relevant event had a similar delay between confirmation of diagnosis and referral for AVR (mean [SD] delay, 27 [34] days vs 20 [33] days, *P* = .58) and comparable rates of New York Heart Association functional class of 3 or more (indicating marked symptoms during daily activity) at baseline (60% vs 41%, *P* = .31).

**Figure.**
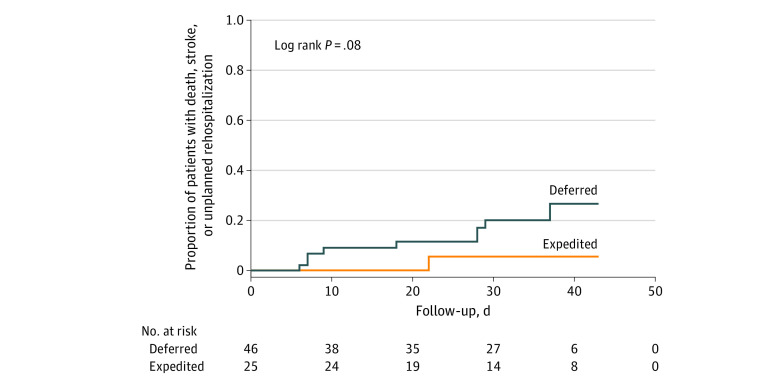
Kaplan Meier Curve of the Primary Composite End Point of All-Cause Death, Disabling or Nondisabling Stroke, and Unplanned Hospitalization for Valve-Related Symptoms or Worsening Heart Failure

## Discussion

The algorithm used in this study to allocate the treatment strategy was similar to the triage recommendation from the American College of Cardiology and Society for Cardiovascular Angiography & Interventions consensus statement issued after initiation of patient recruitment for the study.^3^ Deferral of AVR in patients with symptomatic severe aortic stenosis was associated with an increased risk of hospitalization for valve-related symptoms or worsening heart failure. Patients with symptomatic severe aortic stenosis in combination with relevant multivalvular disease may particularly benefit from expedited AVR. Study limitations include the low patient number owing to the short duration of the ban of elective procedures and the locally modest numbers of severe acute respiratory syndrome coronavirus 2 (SARS-CoV-2) infections, which did not exceed available health care resources.

## Notes

Supplement. **eAppendix.**
