# Supplementary material for: Mortality, Stroke, and Hospitalization Associated With Deferred vs Expedited Aortic Valve Replacement in Patients Referred for Symptomatic Severe Aortic Stenosis During the COVID-19 Pandemic
Source: JAMA Netw Open. 2020 Sep 30;3(9):e2020402. doi: 10.1001/jamanetworkopen.2020.20402 (PMC7527876; doi:10.1001/jamanetworkopen.2020.20402)
Supplement: Supplement. — eAppendix. [file jamanetwopen-e2020402-s001.pdf]

Supplementary Online Content

Ryffel C, Lanz J, Corpataux N, et al. Mortality, stroke, and hospitalization associated with deferred vs expedited aortic valve replacement in patients referred for symptomatic severe aortic stenosis during the COVID-19 pandemic. *JAMA Netw Open.* 2020;3(9):e2020402. doi:10.1001/jamanetworkopen.2020.20402

eAppendix.

This supplementary material has been provided by the authors to give readers additional information about their work.

© 2020 Ryffel C et al. *JAMA Network Open.*

eAppendix

Eligibility criteria and treatment algorithm

All patients with symptomatic severe aortic stenosis defined by an aortic valve area (AVA) ≤1.0 cm^2 or an AVA indexed to body surface area <0.6cm^2/m^2, including low-flow severe aortic stenosis defined by an indexed stroke volume ≤35ml/m^2, that were referred for AVR between March 20 and April 26, 2020 were consecutively included. Patients with a life expectancy <1 year irrespective of valvular heart disease or severe dementia were excluded. The treatment algorithm according to pre-specified criteria is illustrated in Suppl. Figure 1.

Data collection

Baseline and follow-up data were recorded from documentation of referring physicians and standardized telephone interviews. Sweep follow-up by use of telephone interviews was performed between April 23 and April 30, 2020.

Definitions

Deferred treatment was defined as postponement of AVR by three months after referral. Expedited treatment was defined as AVR within 2 week from referral. The primary endpoint was a composite of all-cause mortality, disabling and non-disabling stroke and unplanned hospitalization for valve-related symptoms or worsening heart failure by intention-to-treat as assessed at the end of the Swiss Federal Council's ban of elective procedures and the resumption of routine clinical work. Multivalvular disease was defined by the combination of aortic stenosis with moderate or severe stenosis or regurgitation of at least one additional valve.

Statistical Analysis

Baseline characteristics and clinical findings are presented as numbers and frequencies (%) for categorical variables and as means (± standard deviation) for continuous variables. Categorical variables were compared by means of the Fisher's exact or Chi-square tests, continuous ones by two-

© 2020 Ryffel C et al. *JAMA Network Open.*

sample Student`s t-tests. Time-to-event analyses were conducted using Kaplan-Meier estimates and compared with the log-rank test. All statistical analyses were performed with SAS software (version 9.4).

© 2020 Ryffel C et al. *JAMA Network Open.*

SUPPLEMENTARY FIGURE LEGENDS

Suppl. Figure 1 **Treatment algorithm**

AVR=aortic valve replacement; AVA=aortic valve area

Suppl. Figure 2 **Flowchart according to STROBE statement**

The flowchart illustrates treatment allocation and outcome assessment.

© 2020 Ryffel C et al. *JAMA Network Open.*

SUPPLEMENTARY FIGURES

Figure 1

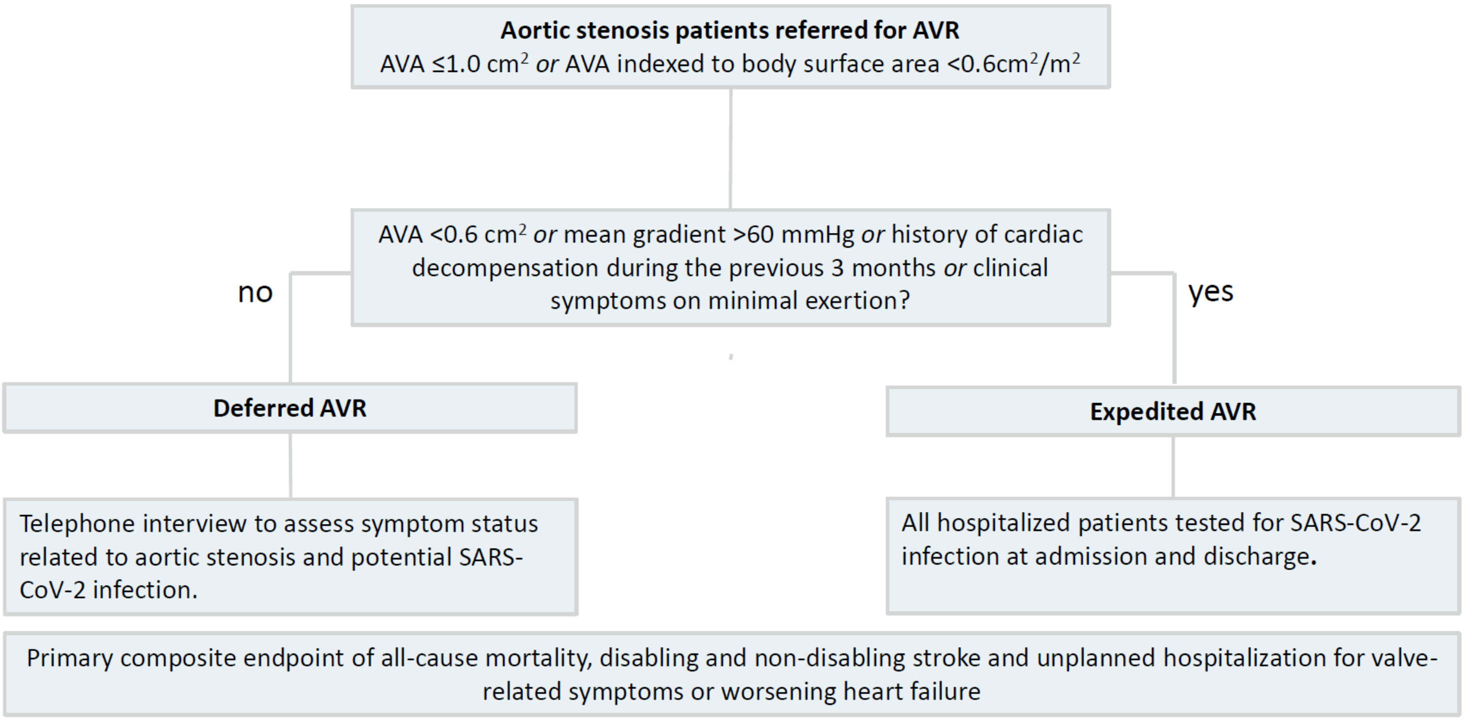

© 2020 Ryffel C et al. *JAMA Network Open.*

Figure 2

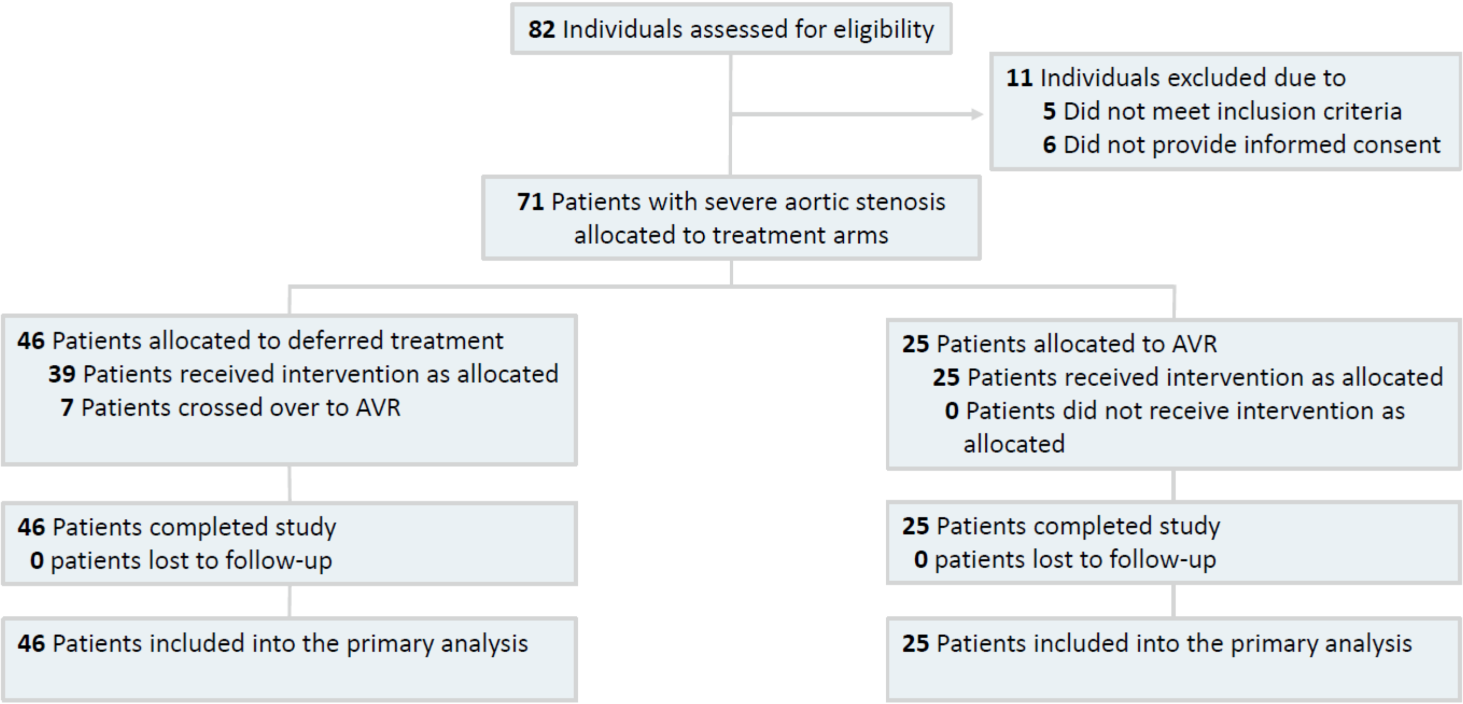

© 2020 Ryffel C et al. *JAMA Network Open.*
